# Medicinal potential of embelin and its nanoformulations: An update on the molecular mechanism and various applications

**DOI:** 10.22038/ijbms.2024.77888.16850

**Published:** 2024

**Authors:** Asad Ali, Nasr A. Emad, Niha Sultana, Hamad Ali, Samreen Jahan, Mohd Aqil, Mohd Mujeeb, Yasmin Sultana

**Affiliations:** 1 Department of Pharmaceutics, School of Pharmaceutical Education and Research, Jamia Hamdard, New Delhi-110062, India; 2 Department of Phytochemistry and Pharmacognosy, School of Pharmaceutical Education and Research, Jamia Hamdard, New Delhi-110062, India

**Keywords:** Embelin, Nanoparticles, Pharmacokinetics, Pharmacologic actions, Structure-activity - relationship

## Abstract

Natural herbs have garnered significant research recently as their components target multiple disease signaling pathways, making them highly potential for various disease prevention and treatment. Embelin, a naturally occurring benzoquinone isolated from *Embelia ribes*, has shown promising biological activities such as antitumor, antidiabetic, anti-oxidant, and antimicrobial. Various mechanisms have been reported, including monitoring genes that synchronize the cell cycle, up-regulating multiple anti-oxidant enzymes, suppressing genes that prevent cell death, influencing transcription factors, and preventing inflammatory biomarkers. However, the hydrophobic nature of embelin leads to poor absorption and limits its therapeutic potential. This review highlights a wide range of nanocarriers used as delivery systems for embelin, including polymeric nanoparticles, liposomes, nanostructured lipid carriers, micelles, nanoemulsion, and metallic nanoparticles. These embelin nanomedicine formulations have been developed in preclinical studies as a possible treatment for many disorders and characterized using various *in vitro, ex vivo*, and *in vivo* models.

## Introduction

**Figure 1:**
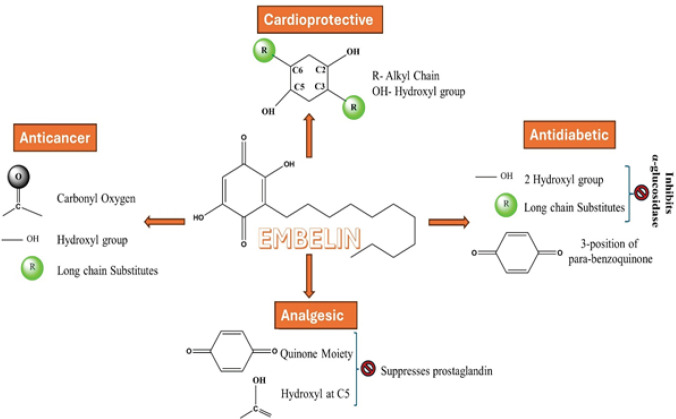
Structure-activity relationship (SAR) of embelin

Embelin (EMB) is a benzoquinone derived from plants that serves as the primary active compound found in the fruits of *Embelia ribes *Burm* (E. ribes)*(1). This species is of Indo-Malaysian origin and is extensively utilized in many conventional drug systems to treat various disorders. EMB is extracted majorly from seeds and pericarp (outer layer) of the *E. ribes* fruit using different solvents such as aqueous solvents, methanol, ethanol, chloroform, acetone, hydroalcoholic solutions, and other organic solvents. The EMB content of *E. ribes* fruits is around 4.33 % w/w when extracted using 100% methanol (2) and 1.86 % w/w using chloroform (3). Various natural compounds, such as quinone derivatives, are recognized for their activity against various disorders due to their superior safety and effectiveness (4). The fruits of *E. ribes*, rich in EMB, a vivid orange hydroxybenzoquinone compound, have gained popularity in ethnomedicine (5). Discovering novel herbal alternatives is crucial since currently accessible drugs are losing their significance in treating various illnesses. EMB can potentially manage cardiovascular disorders (6) and cancers (7, 8). The suppression of Plasminogen Activator Inhibitor-1 (PAI-1) by EMB may account for its impact on various metabolic processes. These include wound healing, blood coagulation, attachment and detachment of cells, cell migration, angiogenesis, and tumor-cell invasion. The structure-activity relationship (SAR) analysis of EMB (Figure 1) revealed that its capability to inhibit PAI-1 is primarily dependent on the presence of hydroxyl groups at positions C_2_ and C_5_, as well as the length of the alkyl chains at positions C_3_ and C_6_ (9). Furthermore, the EMB molecule’s hydroxyl groups, carbonyl oxygen, and lengthy aliphatic chain interact with the peptide backbone and the side chains of different residues. This connection leads to the inhibition of P300/CBP-associated factor (PCAF), resulting in its anticancer effects (10, 11). Moreover, the existence of a quinoline structure with electron-withdrawing groups has a significant impact on preventing myocardial damage and offering cardioprotective benefits (12). Over the last decade, multiple research investigations have shown the antidiabetic properties (13) of EMB and its beneficial impact on blood glucose levels, Hemoglobin Alc levels, insulin levels, and lipid profiles. The SAR investigation indicated that long-chain substituents and two hydroxyl groups were crucial in influencing the inhibitory effect towards α-glucosidase and imparting anti-diabetic benefits.The docking results show that the inhibitory mechanism is based on hydrogen bonds formed between hydrophilic groups and certain amino acids at the active site. Furthermore, it depends on the hydrophobic interactions between the hydrophobic substituents at the 3-position of para-benzoquinone and the hydrophobic pocket (14). EMB has greater scavenging activity for superoxide than commonly used food additive anti-oxidants such as butylated hydroxytoluene and 2,6-bis (1,1-dimethylethyl)-4-20 methyl phenol. EMB, with its significant anti-oxidant properties (15, 16), has the potential to serve as a valuable pharmacological tool for mitigating the damage associated with metabolic and neurodegenerative conditions, including Parkinson’s (17, 18), Alzheimer’s (19-21), Huntington’s (22, 23), and Multiple sclerosis (24). The findings suggest two distinct mechanisms by which EMB acts as a scavenger. The first mechanism involves π- π interaction; the second process, on the other hand, entails capturing protons within the cells (25). In addition, it is used to treat various neurological disorders, including depression (26). It achieves this by increasing the expression of brain-derived neurotropic factors, reducing the activity of oxidative stress markers, increasing the expression of anti-oxidants, decreasing the expression of pro-inflammatory cytokines, and normalizing the activity of the hypothalamus-pituitary axis (27). EMB also suppresses prostaglandin production or function, responsible for its analgesic effects (28-30). According to SAR investigations, the quinone moiety of EMB and the presence of the phenolic OH group at C-5 are essential to its activity. Evidence demonstrates that it impedes the production of pro-inflammatory cytokines, specifically tumor necrosis factor (TNF)-α, interleukin (IL)-1β, and IL-6. This inhibition contributes to decreased organ inflammation and associated damage in conditions characterized by inflammation. The anti-inflammatory effect (31, 32) of EMB is greatly enhanced by the presence of a p-sulfonamide nucleus in its molecular structure, as shown by its SAR investigations (30). Other uses of EMB include anthelminthic (33), carminative(34), diuretic (34), epilepsy (35), traumatic brain injury (34), anxiety (36), cerebral ischemia (37, 38), wound healing (39), anti-bacterial (40, 41), and anti-protozoal effects (42). Because of its many biological uses, EMB is called the “second solid gold of India” after curcumin (43).

Despite plant extracts’ impressive in vitro bioactivity and their phytoconstituents, their *in vivo *activities are constrained by their large molecular dimension and low lipid solubility. As a result, they are less readily absorbed and have poor bioavailability (44). Phytotherapeutics demands an empirical approach to consistently administer its components, ensuring patient adherence and minimizing the need for frequent administration. This could be achieved by developing innovative nanoparticle drug delivery systems (NDDS) for Phyto-therapeutics. NDDS reduce the need for frequent dosing to address non-compliance and enhance therapeutic efficacy by minimizing toxicity, enhancing allocation within tissues (45), and improving bioavailability via increased solubility and permeability (46). Several nanocarriers have been developed and acknowledged for delivering herbal phytoconstituents. These include organic nanoparticles (NPs) such as liposomes, nanoemulsions, niosome, self-nanoemulsifying drug delivery systems (SNEDDs), transfersomes, nanostructured lipid carriers (NLC), solid lipid Nanoparticles (SLN), and phytosomes. Inorganic NPs like silver NPs, gold NPs, zinc oxide NPs, carbon nanorods, and quantum dots are also used. Additionally, polymeric NPs such as chitosan NPs, micelles, polyglutamic acid (PGA), polylactic acid (PLA), poly (lactic-glycolic acid) (PLGA), and dendrimers are employed (47, 48). Nevertheless, the effectiveness of often-used herbal monotherapies in preventing the progression of diverse conditions and alleviating their multiple symptoms is insufficient. Combination therapy is gaining attention because of the potential synergistic effects arising from various phyto-therapeutics’ diverse mechanisms of action.

## Materials and Methods

A comprehensive search was conducted across many electronic databases, including PubMed/Medline, Springer, Scopus, SciFinder, ScienceDirect, and Google Scholar, using keywords such as embelin, nanoparticles, combination nanocarriers, structure-activity relationship, bioavailability, pharmacokinetics, and pharmacological effects. This study provides an extensive literature analysis of various research conducted on embelin, focusing specifically on its antidiabetic, cardioprotective, neuroprotective, anti-inflammatory, antiviral, antibacterial, anti-oxidant, wound healing, antifertility, analgesic, and anticancer properties. This study also showcases a diverse array of nanocarriers used as delivery vehicles for embelin, including polymeric nanoparticles, liposomes, nanostructured lipid carriers, micelles, nanoemulsion, and metallic nanoparticles. Qualified articles were chosen for this review and discussed per the literature search parameters.

**Figure 2:**
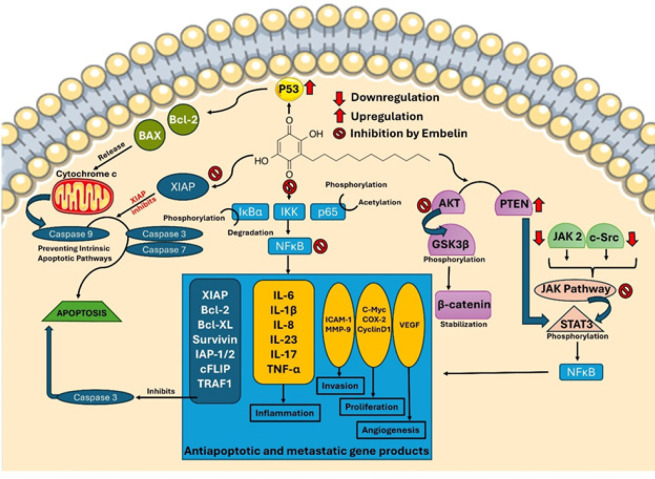
Role of Embelin (EMB) in cancer treatment

Bcl-2: B-cell lymphoma 2, Bcl-XL: B-cell lymphoma-extra-large, BAX: Bcl-2-associated X protein, AKT: Protein kinase B (PKB), PTEN: Phosphatase and TENsin homolog deleted on chromosome 10, JAK: Janus kinase/signal transducers and activators of transcription, c-Src: cellular sarcoma kinase, c-Myc: cellular Myc, GSK3β3: Glycogen synthase kinase 3, VEGF: Vascular endothelial growth factor, STAT3: Signal transducer and activator of transcription 3, P53: tumor protein, ICAM-1: intercellular adhesion molecule, IkBa: NF-κB inhibitor alpha, IKK: inhibitor of nuclear factor-κB (IκB) kinase, NFkB: Nuclear factor kappa B, MMP-9: Matrix metalloproteinase

**Figure 3:**
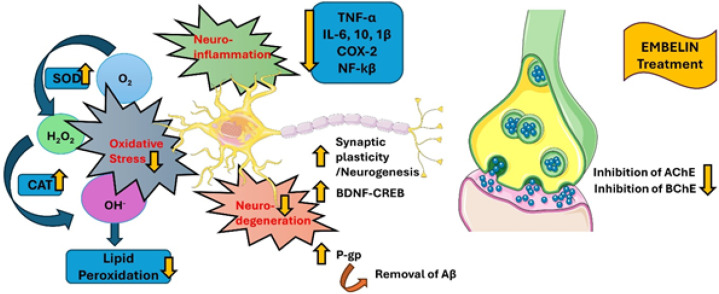
Effect of Embelin (EMB) on various inflammatory and oxidative stress markers

SOD: superoxide dismutase, OH-: Hydroxide ion, CAT: catalase, TNF-α: Tumor necrosis factor alpha, COX-2: Cyclooxygenase 2, IL: Interleukin, AChE: Acetylcholinesterase, BChE (Butyrylcholinesterase), Amyloid Protein (Aβ)

**Figure 4:**
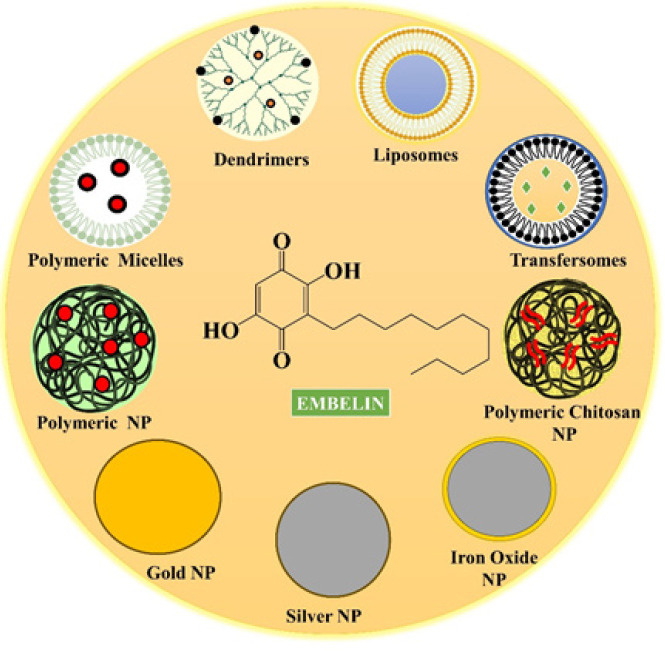
Various nanoformulations used in literature for Embelin (EMB) delivery

**Table 1:** Nanocarriers for Embelin (EMB) delivery in several diseases

Embelin nanocarriers	Disease	Findings and need	Flaws of nanocarriers	Reference
Liposomes	Depression	-Smaller particle size, increased surface area, and solubility. -Enhanced permeation and biodistribution. -Sustained drug release and enhanced biocompatibility	-Clearance by the immune system, particularly by macrophages -Low drug loading, high cost, and stability challenges
NLCs	Epilepsy	-Sustained release, minimized drug leakage, and brain targeting through nasal route -High drug entrapment and prevent lipid crystallization which leads to a more flexible and disordered lipid structure	-Endosomal escape challenges and short blood circulation time -NLCs can exhibit instability under *in vivo* conditions
Transfersomes	Skin Cancer	-High potential as a transdermal drug delivery system for the treatment of skin cancer -Transfersomes have enhanced transdermal flux, entrapment, sustained release, and minimized side effects -Transfersomes are highly elastic and ultra deformable	-Higher cost, manufacturing complexity, and limited scalability -Stability concerns during storage and transport
Lipid Nanospheres	Ulcerative colitis	-Narrow polydispersity and highly negative charged particles showing excellent stability -Improved *in vivo* efficacy and sustained release -Nanospheres provide excellent drug protection and targeted drug delivery	-Chances of potential aggregation, instability issues during storage, and cytotoxicity concerns -Have low drug loading capacity and limited drug release control
Chitosan NPs	Arthritis	-Significantly reduced paw swelling and arthritic score. - Inflammatory markers reduced to normal levels in a dose-dependent manner -Reduced toxicity, increased biodegradability, have positive surface charge, and can be used in oral, nasal, pulmonary, and mucosal drug delivery	-High hydrophilicity, solubility issues at certain pH, chances of potential aggregation, and limited drug encapsulation
PLGA NPs	Hepato-toxicity	-Higher cellular uptake in Hep G2 cells and better biodistribution -Higher stability, improved water solubility, high drug loading capacity, enhanced drug localization in target sites, sustainable drug release, and the ability to optimize drug biodistribution while reducing toxicity	-Challenges in drug penetration and drug loading capacity
Gold NPs	Multidrug resistance bacterial infections	-Reduced the minimal inhibitory concentration and increased antibacterial efficacy by 4 folds showing a synergistic effect -Useful in medical imaging and hyperthermic treatment -Biocompatible, higher stability, targeted administration, and easy modification to include functional groups like targeted antibodies	-Synthesis of Gold NPs can be complex and may involve multiple steps -Gold NPs may raise toxicity concerns, especially related to their potential impact on biological systems and the environment
Silver NPs	Lung cancer	- Smaller size (25 nm) and negatively charged particles indicating higher stability -Considerable dose-dependent suppression of A549 cell line growth - Silver NPs have intrinsic anti-inflammatory, antioxidant, antibacterial, antiviral, and antifungal activities	-AgNPs can migrate from the lungs to other organs such as the liver, kidney, or brain, which may lead to systemic effects and impact the functioning of essential organs -Propensity to create harmful radicals which damage DNA, proteins, and membranes -AgNPs may enter the brain and cause neuronal degeneration and necrosis, affecting brain health and cellular function

**Table 2:** Combination nanoformulations of embelin (EMB) with other therapeutic moieties

Nanoformulation/ Route of administration	Combination drugs/ DOSE	Results/ Superiority	Disease	Reference
PLGA–chitosan core–shell NP/ Oral administration	EMB and RPI-1 (indolinone derivative) (1:4.7)	Findings suggest that the substance is biocompatible, exhibits a particular affinity for PC cells, and has the potential to induce cell death and impede metastasis effectively.	Pancreatic cancer
Liposomes (conjugated with transferrin)/ Oral administration	Doxorubicin and EMB	Showed smaller particle size, uniform and spherical morphology, and higher entrapment. Potent inhibition of breast cancer cells.	Breast cancer
SLNs/ Parenteral administration	Paclitaxel and EMB/ 6 µg per ml (IC50)	Particle size around 300 nm, higher entrapment, and *in vitro* drug release. Higher cell toxicity in MCF-7 breast cancer cell line	Breast cancer
SNEDDs/ Oral administration	EMB (30mg/kg) and Gliclazide (10mg/kg)	Particle size less than 200 nm, low PDI, high entrapment, better release as compared to suspension, and highly negative zeta potential indicating higher stability. They are treating streptozotocin-induced hyperglycemia.	Diabetes
pH-sensitive amphiphilic polymeric NPs/ Parenteral administration	EMB (1.33 mg) and tumor necrosis factor-related apoptosis-inducing ligand plasmid (pTRAIL)	MDA-MB-231 TNBC cells exhibit enhanced drug uptake compared to MCF-7 non-TNBC cells which have lower CD44 expression. It enhanced cytotoxic and pro-apoptotic effects. Elevation in ROS levels and suppression of the expressions of proteins associated with apoptosis.	Triple-negative breast cancer (TNBC)
NLCs/ Intranasal administration	Donepezil hydrochloride and EMB (1:1)	Smaller particle size, i.e., below 200 nm, higher stability, and controlled drug release. Higher drug uptake in N2a cells and better brain targeting using the nasal route.	Alzheimer’s disease
Chitosan gold NPs	EMB and ciprofloxacin	NPs decreased the minimum inhibitory concentration of ciprofloxacin by 16-fold and 4-fold against multiple drug-resistant strains. Fractional inhibitory concentration confirmed the synergy between the EMB Chitosan gold NPs and Ciprofloxacin.	Antibiotic resistance
Micelles/ Parenteral administration	Paclitaxel (20 mg/kg) and EMB	Micelles show gradual release of the drug over 5 days. The combination of polyethylene glycol and EMB forms stable micelles in water and effectively encapsulates the hydrophobic drug paclitaxel.	Breast and prostate cancer
Chitosan NPs/ Intranasal administration	EMB and carbidopa	NPs exhibited sustained release patterns for 10 hr. Gamma scintigraphy imaging in rats demonstrated a significant increase in the amount of EMB with carbidopa-loaded chitosan NPs that reached the brain, resulting in enhanced bioavailability.	Parkinson’s disease

## Results

***Embelin pharmacokinetics and bioavailability***

Improving our understanding of bioavailability and conducting relevant human studies can be achieved by the discovery of pharmacokinetic properties of bioactive compounds. Because of its poor bioavailability caused by its low solubility and high permeability after oral administration, EMB is classed as a class II drug in the biopharmaceutics classification system (BCS). According to pharmacokinetic studies performed by Li *et al*., the elimination process of EMB was rapid, as shown by the half-life (t_1/2_) of 1.52±0.83 hr after intravenous administration at a dose of 5 mg/kg. The level of EMB in rat plasma exhibited a sharp decline during the first 15 min, followed by a gradual reduction. The AUC_0-24_, AUC_0-∞_, and MRT_0-24 _values were 2.17±0.49 μg/ml h, 3.21±0.62 μg/ml h, and 1.28±0.11 h, respectively. Whereas, on oral administration of EMB at a dose of 15 mg/kg it showed a T_max_ value of 0.31±0.18 h indicating that embelin reaches its highest plasma concentration rapidly and half-life of 1.01±0.58 hr, indicating a rapid elimination process. The plasma concentration of embelin was found to be low, and the absorption was incomplete. This was evident from the volume of distribution of more than 6 L and the C_max_ value of 1.04±0.21 μg/ml, which was much lower than the C_max_ value of 3.91±1.34 μg/ml seen with intravenous administration. This outcome was attributed to the intrinsic low water solubility of embelin. The AUC_0-t_ value was 1.97±0.78 μg/ml h, the AUC_0-∞_ value was 2.92±0.69 μg/ml h, and the MRT_0-t _value was 1.49±0.49 h (49). The oral bioavailability of embelin is very limited, that is 30.2±11.9% (49). EMB’s limited applicability is mostly due to its poor water solubility leading to its lower bioavailability. As a result, researchers have investigated several methods of delivering drugs to enhance the capacity of embelin to dissolve in water, as well as its stability and effectiveness. One such method includes the development of nanocarriers for drug delivery as discussed in the later section of nanoformulations of EMB. Other methods include encapsulation of drugs such as one by Li *et al*. who synthesized a copolymer specifically designed to improve the ability of embelin to dissolve in polymeric micelles. The investigators conducted a study where they attached a dodecanol lipid chain to a poly (ethylene glycol)-b-polycarbonate (PEG-b-PBC) copolymer backbone and investigated how varying chain lengths affected the loading efficiency. An increase in embelin loading efficiency was found, going from 40% in PEG-b-PBC micelles to 100% in poly (ethylene glycol)-block-poly (2-methyl-2-carboxylpropylene carbonate-graft-dodecanol)(PEG-b-PCD) lipopolymer (50). Using a different approach, Li’s team enhanced the water solubility of embelin by linking it to PEG 3.5K by conjugation. The conjugates of PEG3.5K and embelin exhibited an aqueous solubility above 200 mg/ml (51). Various analogs of embelin have also been chemically produced to enhance their water solubility and improve their effectiveness as medicinal agents (52). Singh and his colleagues developed embelin derivatives to tackle the problem of low solubility in water. They incorporated nitrogen-containing heterocycles into the embelin scaffold and synthesized hydrochloric acid salts of the resulting derivatives. The Mannich reaction was used to include N-linked functionalities to enhance the hydrophilicity of embelin (53).

***Biological activity of EMB in various diseases***

EMB is well known for its broad spectrum of biological activities that possess potential against cancer, CNS disorders, diabetes, malaria, microbes, etc. It also has anti-oxidant and anti-inflammatory activities which can be further used in various treatment strategies.

*Anti-cancer*

EMB, a naturally occurring molecule, has potential anticancer properties. It has demonstrated a wide range of biological actions, including anti-inflammatory, anti-oxidant, and anticancer properties. A key mechanism involves targeting multiple signaling pathways crucial for cancer cell proliferation, survival, and metastasis (Figure 2). EMB inhibits the activity of nuclear factor-kappa B (NF-κB), a transcription factor that plays a crucial role in regulating the expression of genes associated with inflammation, cell survival, and proliferation. This inhibition occurs through the mechanism of NF-κB inhibition. NF-κB is often overactivated in cancer cells, contributing to their uncontrolled growth. By blocking NF-κB, EMB helps in suppressing the growth and survival of cancer cells (54). EMB also possesses anti-angiogenic properties, i.e., it can inhibit angiogenesis. Disrupting angiogenesis limits the blood supply to tumors, thereby impeding their progression (55). EMB has demonstrated the ability to induce apoptosis, which is a regulated form of cell death, in different types of cancer cells. Apoptosis is another critical mechanism that EMB follows to eliminate abnormal and damaged cells, preventing the uncontrolled proliferation of cancer. The apoptotic effect of EMB involves the activation of caspases and modulation of Bcl-2 family proteins (56). 

*Neuroprotective*

EMB shows a neuroprotective effect by inhibiting amnesia and enhancing learning and memory in Alzheimer’s disease. It serves as a multi-targeted moiety, diminishing amyloid oligomer formation by inhibiting clinically recognized targets such as beta-secretase (BACE-1 (beta-site amyloid precursor protein cleaving enzyme 1)) and cholinesterase. Furthermore, it improves the transmission of cholinergic signals by inhibiting the enzymes AChE (Acetylcholinesterase) and BChE (Butyrylcholinesterase), and it facilitates the removal of amyloid proteins (Aβ) by stimulating the production of P-gp (57)(Figure 3). EMB significantly affects neuronal damage caused by temporary global ischemia/reperfusion in rats. The group that received EMB exhibited a substantial rise in locomotor activity, a marked decrease in lipid peroxidation, and an elevation in both the overall thiol content and glutathione S-transferase activity in brain homogenates. Furthermore, the cerebral effects of EMB were evident in the reduced area of cerebral infarction and significant histopathological improvements, including normal glial density, reduced edema, the absence of lymphocytes, alleviated blood vessel congestion, and a decrease in necrosis (37). These findings collectively suggest that EMB exhibits potent neuroprotective properties and could prove beneficial as an adjunct in the treatment of cerebral stroke. EMB is seen to have a therapeutic potential against depression by reducing immobility in rat models (58). 

*Anti-diabetic*

The EMB quinone derivative decreases the levels of insulin in the plasma by inducing the movement and activation of GLUT4 (Glucose transporter type 4) in the PI3K/p-AKT (phosphoinositide 3-kinase/ Protein kinase B) signaling pathway. EMB has stable binding interactions with key active sites such as PI3K, p-AKT, and GLUT4 (59). EMB has demonstrated regenerative activity on islet cells when introduced to the degenerated pancreas. This property helps protect the β-cells against loss, showcasing anti-diabetic properties (60). Anti-obesity potential of EMB is also revealed. It exhibited preventive activity on weight gain, accumulation of visceral fat, and higher blood pressure (61). The aqueous extracts derived from *E. ribes* showed an improvement in insulin resistance in a rat model with obesity produced by a high-fat diet. The potential mechanism underlying this improvement involves the inhibition of gene expressions associated with leptin, tumor necrosis factor-alpha (TNF-α), sterol regulatory element-binding proteins 1 gamma (SREEBP1), and peroxisome proliferator-activated receptor gamma 2 (PRARγ2). Leptin may have a role in the development of hepatic steatosis by promoting insulin resistance and altering insulin signaling in liver cells, leading to an increased accumulation of fatty acids inside the cells (62). 

*Cardioprotective*

The cardioprotective action of EMB involves interrupting mitochondria-dependent apoptotic damage, increasing myocardial cytochrome c, cleaved-caspase-3 & 9, and PARP (Poly (ADP-ribose) polymerase) levels, as well as restoring succinate dehydrogenase, cytochrome c oxidase, and mitochondrial redox activity. EMB also strengthens anti-oxidant status, attenuates myocardial lipid peroxidation, decreases cardiac injury markers, and exhibits histopathological benefits (63). This natural compound shows promise in preventing ischemic heart diseases like myocardial infarction. Additional studies highlight the potential cardioprotective effects of embelin against myocardial injury, emphasizing its role in reducing oxidative stress, apoptosis, and cardiotoxicity, thus suggesting its therapeutic benefits in heart conditions (64).

*Wound Healing*

EMB has shown considerable promise in augmenting the mechanisms involved in wound healing. Research investigations have shown that embelin enhances the healing of cutaneous wounds by raising the density and strength of granuloma tissue in models such as the dead space model (65). In addition, EMB has strong antiangiogenic properties, which further confirms its involvement in wound healing by blocking mitochondrial respiration in endothelial cells (66). EMB has been seen to have a beneficial effect on the healing of skin wounds in rats with diabetes. This is supported by the greater rates of wound contraction and the higher levels of hydroxyproline in the treated animals (67). These data demonstrate that embelin has positive effects on facilitating wound healing and indicate its potential use in treating chronic wounds. The diverse effects of embelin in promoting wound healing highlight its therapeutic significance and open up avenues for future investigation into its mechanisms and clinical uses in the area of wound care.

*Anti-oxidant and anti-inflammatory activity*

The presence of a hydroxyl group is beneficial in the investigation of radical scavenging abilities due to its ability to liberate a hydrogen atom, thereby diminishing the reactivity of the scavenged radical. EMB has shown that it can get rid of reactive oxygen species like hydrogen peroxide and superoxide anion radicals by taking electrons and releasing molecular oxygen in a unique way (14). Free radicals are known to damage cell membranes called lipid peroxidation. By preventing lipid peroxidation, EMB helps maintain the integrity of cell membranes and protects cells from oxidative damage (68). EMB augments the cellular anti-oxidant defense system by elevating the activity of enzymes such as superoxide dismutase and catalase (69). Alongside the anti-oxidant activity, EMB also possesses anti-inflammatory activity. The potential mechanism is the mitigation of oxidative stress and inflammation by inhibiting the NF-κB pathways (70). 

*Antibacterial and antiviral activity*

EMB renders a potential activity against various microorganisms such as bacteria and viruses. EMB has potential as an antibacterial agent as suggested by the study where it showed an inhibitory effect against *Staphylococcus aureus* (41). Antiviral activity has also been reported against the influenza virus (71). Additionally, a study of EMB’s molecular docking shows that it binds to the viral hemagglutinin receptor-binding site. This shows that it can stop virus reproduction in the early stages of the viral life cycle. In a recent study (2020), the inhibitory mechanism of the quinone derivative EMB, alongside two potential compounds, methylprednisolone and dexamethasone, against SARS-CoV was elucidated through computational studies (72).

*Anthelmintic*

EMB has shown notable anthelmintic efficacy in several research investigations. Studies conducted on the Ethiopian medicinal plant *Embelia schimperi* have demonstrated that embelin, in both its crude hydroalcoholic extract form and as a diammonium salt, possesses anthelmintic properties against intestinal parasites such as the dwarf tapeworm and hookworm. Notably, it has shown significant rates of parasite clearance in both *in vivo* and *in vitro* models (73). Furthermore, research on the seeds of *E. ribes* has shown the strong anthelmintic effects of aqueous and alcoholic extracts against parasites such as *Haemonchus* *contortus* in sheep. This demonstrates the effectiveness of embelin in fighting helminthic infections (74). Researchers have conducted investigations on the anthelmintic properties of *E. schimperi* from Tanzania, which provide more evidence supporting the traditional use of this plant for its ability to treat parasitic worm infections (75). In a separate study, a combination of an aqueous alcoholic extract derived from the fruits of *E. ribes* and the seeds of *Vernonia anthelmintica* resulted in a significant reduction in the number of eggs found in the feces. The improvement in hematological and biochemical data indicated a decrease in the number of worm infestations. The coprological study revealed that *H. contortus* was the most prevalent kind of strongyle. Therefore, the formulation demonstrated significant effectiveness against *H. contortus*, the most harmful parasite in small ruminants, which has shown resistance to many anthelmintic drugs (76). 

***Nanoformulations of Embelin***

*Lipid-based nanoparticles (Organic)*

Nanotechnology has dramatically impacted the biotechnology, food, cosmetics, and pharmaceutical sectors. Over 40% of drugs that have been approved have low solubility and have a lipophilic nature. Solubility is the primary rate-limiting phase affecting a drug’s release profile and bioavailability. Researchers have identified several methods to administer lipophilic molecules with increased bioavailability and solubility. Nanotechnology is essential for the targeted distribution of poorly soluble drugs. Since they employ biocompatible and biodegradable lipids, lipid-based nanocarriers have attracted a lot of attention in the last 20 years. Lipid nanocarriers are preferred to polymeric NPs. The problems that polymeric NPs have, such as cytotoxicity and a lack of practical large-scale manufacturing techniques, may be solved by lipid NPs (77, 78). The most common lipid-based NPs utilized in pharmaceutical formulations are solid lipid nanoparticles, liposomes, nanoscale lipid carriers, nanoemulsions, and lipid polymer hybrid NPs. Other lipid-based NPs majorly used to deliver phytoconstituents include phytosomes, niosomes, transfersomes, transniosomes, and ethosomes (Figure 4 and Table 1)(79). 

Liposomes are widely used and extensively studied nanocarriers due to their inherent safety characteristics. They function as artificial equivalents of natural membranes. Phytosomes, also called herbosomes, are complexes of phyto phospholipids that have been used recently to enhance the bioavailability of certain insoluble phytoconstituents and plant extracts. Phytosomes demonstrate superior absorption and permeability through membranes compared to regular herbal extracts. Liposome and phytosomes techniques may be advantageous in delivering various phytoconstituents and herbal extracts to target the treatment actively and passively and/or imaging a wide range of disorders (88). EMB-loaded liposomes were created and optimized for intranasal delivery in the treatment of depression in research conducted by Ali *et al*. The optimized nanoliposomes demonstrated vesicle sizes of less than 100 nm, high dispersity, increased drug release, and good entrapment efficiency. Greater anti-oxidant capacity than standard ascorbic acid was discovered. Studies on *ex vivo* permeation revealed that produced liposomes had far higher penetration than the EMB solution. Additionally, rhodamine B-loaded liposomes seemed to have greater penetration in the nasal mucosa when compared to the control group (rhodamine B-solution) using Confocal laser scanning microscopy. Based on all the experimental results, it was determined that liposomes loaded with EMB were a viable and efficient formulation for intranasal administration and could be further evaluated for their anti-depressant efficacy (80). In a separate study, Yadav *et al*. synthesized the EMB-loaded phytosomes complex to investigate the potential protective effects against paracetamol-induced hepatic damage in male Wistar rats. Phytosomes showed minimum particle size, less dispersity, higher entrapment, and increased *in vitro* drug release. EMB phytosomes exhibited hepatoprotective effects in paracetamol-induced hepatotoxicity comparable to standard silymarin (89).

In a separate investigation, Alam *et al*. developed an oral niosome formulation of EMB utilizing a thin-film hydration process and then examined its antidiabetic efficacy. The study found that the niosomes containing EMB exhibited superior encapsulation efficiency, vesicle size, lesser polydispersity index (PDI), and better *in vitro* release than EMB in its free form. The *in vivo* study of optimized formulation in Wistar rats demonstrated a hypoglycaemic effect like that of the conventional antidiabetic medication, repaglinide. Furthermore, the evaluated biochemical parameters showed favorable anti-oxidant activity of the noisome formulation. Therefore, niosome formulation loaded with EMB effectively managed diabetes in Wistar rats (90).

A significant obstacle in delivering large natural phytoconstituents is their loading in lipid vesicles. One possible method to address the challenge is loading large molecular weight constituents in transfersomes. Their structure consists of two layers, allowing for the efficient containment of both lipophilic and hydrophilic drugs and drugs with both properties. This results in greater rates of drug absorption compared to traditional liposomes. Transfersomes possess elastic properties, allowing them to undergo deformation and compression as a whole vesicle, enabling them to pass through small openings much smaller than their size (91). Researchers developed and assessed transfersomal vesicles containing EMB as a transdermal drug delivery method for treating skin cancer. The vesicles exhibited a spherical form, as verified by transmission electron microscopy. Additionally, these vesicles had the highest level of EMB entrapment efficiency. They conclude that it has the potential to serve as a transdermal drug delivery method for treating skin cancer (82).

Another strategy to deliver phytoconstituents involves the use of lipid-based nanospheres. For example, EMB lipid nanospheres were produced by Badamaranahalli *et al.* to enhance the efficacy of ulcerative colitis therapy. The results demonstrated that EMB was released in a controlled manner over time. However, the method by which the medication seemed to be released was non-Fickian diffusion. *In vivo* experiments utilized a rat model of ulcerative colitis caused by acetic acid. The findings demonstrated that treatment with EMB lipid nanospheres substantially decreased macroscopic scores and clinical activity compared to treatment using an EMB conventional solution. The administration of EMB LNs resulted in a reduction in MPO (myeloperoxidase), LDH (lactate dehydrogenase), and LPO (lipid peroxidation) levels, as well as an increase in reduced GSH (glutathione levels). These changes imply that the therapy of ulcerative colitis was improved. This was further validated by enhanced histological conditions. Therefore, researchers concluded that EMB lipid nanospheres could effectively treat ulcerative colitis (83).

Emulsions are colloidal suspensions consisting of microscopic droplets of one liquid phase scattered inside another. Nanoemulsions are emulsions that have been reduced to nano-sized particles, typically ranging from tens to hundreds of nanometres. These emulsions possess highly desirable properties, including small particle sizes, a large surface area relative to their volume, enhanced dispersion of hydrophobic components, and improved absorption. As a result, nanoemulsions hold significant promise for applications in pharmaceutics, foods, and cosmetics (92). It is well-established that many herbal drugs exhibit low solubility, hydrophobic characteristics, and limited distribution. Consequently, their bioavailability is diminished, resulting in lower effectiveness of therapy. This necessitates either repeated administration or higher doses. In recent decades, there has been a strong focus on the advancement of self-emulsifying drug delivery systems (SEDDS) for herbal treatments. SEDDS are stable and homogeneous solutions comprising oil, surfactant, co-surfactant, and drugs. When these solutions are combined with water and gently stirred, they can spontaneously form nanoemulsions with oil droplets dispersed in water. The formulation may be a feasible substitute for the conventional formulations, focusing on their lipophilic properties and addressing issues such as limited solubility, inadequate bioavailability, inadequate oral absorption, and instability (93,94). Researchers fabricated a nanosuspension of EMB intending to enhance the solubility and dissolving characteristics of EMB using a wet media milling technology. In addition, nanosuspensions underwent freeze-drying to produce nanocrystals. The formulations exhibited a substantial improvement in both solubility and dissolving rate. The results indicated a significant enhancement in the solubility characteristics of EMB by the production of nanocrystals (95). In a different study, they developed a solid self-nanoemulsifying drug delivery system (S-SNEDDS) with Capryol-90 as the oil phase to efficiently transport EMB. The SNEDDS *in vitro* dissolving study showed a higher rate of drug dissolution. There was no indication of a physicochemical interaction between the drug and excipients, according to the FT-IR investigation. The solid-state analysis of S-SNEDDS using powder x-ray diffraction and differential scanning calorimetry revealed a decrease in the phytoconstituent’s crystallinity. This finding further strengthens the findings obtained from the dissolution investigations. Current research has shown that porous carriers based on S-SNEDDS can increase the solubility of the weakly water-soluble herbal active component, EMB (96).

Liposomes, nanoemulsions, niosomes, and polymeric NPs are examples of typical colloidal carrier systems that have constraints that are overcome by solid lipid nanoparticles (SLNs) and nanostructure lipid carriers (NLCs). Formulation scientists have shown significant interest in SLNs over the last two decades owing to their compatibility and ability to penetrate various physiological barriers. Solid lipids are becoming more prevalent than liquid oils in developing controlled-release nanoformulations because drug molecules have limited movement inside a solid lipid matrix (97). Utilizing biological lipids in the production of SLNs decreases the likelihood of acute or chronic toxicity. SLNs have several benefits compared to other nanocarriers, including their facile ability to be produced on a large scale, enhanced bioavailability, little chronic or acute toxicity, and the capacity to include hydrophilic and lipophilic medicines. The unique characteristics of SLNs make them superior as drug carriers compared to conventional nanoformulations (98). NLCs are specifically engineered to address the constraints of SLNs, making them the subsequent version of SLNs referred to as the second generation. Colloidal drug carrier systems provide precise drug delivery and enhance hydrophobic medications’ effectiveness while safeguarding delicate active ingredients. NLCs, like SLNs, maintain a solid lipid matrix at room and body temperature. However, they possess distinct internal frameworks in comparison to SLNs (99, 100). Additionally, these nanocarriers find extensive utility in administering herbal medications (101-104). Nanolipid carriers loaded with EMB were produced for brain targeting by Sharma *et al*. Compared to plain EMB and commercial formulation, *in vivo* findings reveal that NLCs deliver a greater concentration of EMB into the brain. The activity of EMB and its justification for usage in treating epilepsy could be better understood with the help of biochemical parameter verification. Compared to plain EMB and commercially available formulations, the intranasal EMB-loaded NLC formulation demonstrated much higher efficacy. It works much the same as the commercially available version. The above evidence suggests that nanolipid carriers loaded with EMB may be an efficient and appropriate vehicle for brain targeting (81). Investigators synthesized sterically stabilized SLNs that included EMB and paclitaxel. These underwent further characterization and *in vitro* cytotoxicity testing. The results indicated that the optimized SLN formulation might be used as an alternate delivery strategy for the parenteral administration of paclitaxel and EMB in the treatment of breast cancer (105).

*Polymeric nanoparticles*

Polymeric NPs have garnered considerable interest in recent years due to their distinctive characteristics resulting from their diminutive dimensions. The advantages of using polymeric NPs as drug carriers include their capacity to regulate the release of medications, protect them from their surroundings, and enhance their effectiveness and safety (106). Herbal-based polymeric NPs are now the leading and developing polymeric nanocarriers, attracting significant scientific interest in innovative medication delivery methods. Polymeric NPs containing herbal extracts such as EMB (Table 1) provide a very effective and new method for delivering herbal drugs to their target site of action, resulting in significant therapeutic benefits. Hence, ongoing comprehensive scientific investigations are being conducted in herbal drug technology, which presents numerous advantages in delivering phytoconstituents to specific locations. This system holds great potential for controlled drug delivery and targeting (107). Kumar *et al.* fabricated surface-modified NPs containing EMB, composed of gallic acid (GA) and PLGA. The conjugated NPs greatly enhanced the absorption of the medication in the liver by 2.5 times more than the pure drug. This may be explained by an increase in the drug uptake by liver cells and the specific targeting of the liver *via* the interaction of GA with its GA receptors in the liver. The NPs exhibited hepatoprotective effects by decreasing the levels of serum glutamic-oxaloacetic transaminase (SGOT), serum glutamic-pyruvic transaminase (SGPT), alkaline phosphatase (ALP), and total bilirubin (TB) in comparison to the alcoholic-hepatotoxic group (85). Additionally, researchers have documented chitosan NPs to serve as a potentially effective vehicle for herbal phytoconstituents, surpassing the capabilities of conventional carriers and enhancing pharmaceutical efficacy(108). Researchers formulated chitosan NPs loaded with EMB to be used to manage rheumatoid arthritis. Treatment with EMB-chitosan NPs resulted in a substantial reduction in arthritic score and paw edema. Additionally, it decreased malondialdehyde and nitric oxide levels, restoring anti-oxidant levels to their baseline levels by alleviating oxidative stress. Administration of chitosan NPs also led to a considerable decrease in serum levels and normalization of inflammatory indicators such as TNF-α, IL-6, and IL-1β (84).

In separate research, Maanvizhi *et al*. constructed EMB-chitosan NPs to treat streptozotocin-induced diabetes. Chitosan NP-treated animals showed a significant decrease in glucose levels compared to diabetic control rats. In addition, histological analysis demonstrated that rats treated with chitosan NPs were not harmful at doses up to 25 mg/kg body weight. The study has shown that EMB-loaded chitosan NPs had anti-diabetic properties and might be advantageous in managing hyperglycemia in individuals (109). In another investigation, N, O-Carboxymethyl Chitosan NPs were produced and loaded with EMB to investigate its anti-oxidant and cytotoxic effects. The produced NPs exhibited a particle size of 650–850 nm and had a strongly negative charge, verifying their long-term stability. NPs demonstrated remarkable anti-oxidant capabilities and significantly inhibited the proliferation of osteosarcoma MG-63 cells. The results showed that the chitosan NPs loaded with EMB effectively delivered EMB to cancer cells (110).

*Metallic Nanoparticles (Inorganic)*

The use of metallic NPs as an innovative therapeutic tool has substantial promise for enhancing the treatment and diagnosis of many disorders. The utilization of metal nanoparticles (MNPs) has garnered significant interest and led to noteworthy advancements, particularly in the medical field. MNPs enhance the effectiveness of drugs by targeting specific sites, overcoming resistance to many treatments, and efficiently delivering therapeutic molecules. In addition to medication administration, MNPs have many other well-known uses in medicine, including the creation of improved biocompatible materials, nutraceuticals, and *in vivo* and *in vitro* diagnostics. Adding metallic NPs to drug delivery systems has many benefits, such as making the drug more stable and extending its half-life in the bloodstream. They can also help with biodistribution and can be passively or actively targeted to a specific area of target. An emerging area in bio-nanotechnology is the green synthesis of MNPs, presenting advantages over chemical and physical processes in terms of both economic and environmental considerations (111,112).

Silver NPs are essential and intriguing nanomaterials among the many metallic NPs used in biomedical applications. Phytoconstituent-mediated silver nanoparticles (AgNPs) are both safe and eco-friendly. They are also cost-effective and can be swiftly produced. They are also cost-effective and can be swiftly produced. Additionally, they serve essential functions as reducing, stabilizing, and capping agents. Hence, the environmentally friendly approach to creating AgNPs has many benefits compared to conventional chemical and physical approaches (113). Researchers synthesized EMB-based AgNPs to treat cancer. The potential of EMB-fabricated AgNPs to inhibit cancer was examined via *in vitro* research on lung cancer cells using the MTT test. The findings demonstrated a notable and gradual decrease in cell growth inhibition with the dosage administered, specifically targeting the A549 cell lines. The apoptosis generated by EMB AgNP was quantified using the annexin-V PI apoptosis test, which showed a much lower number of necrotic cells compared to apoptotic cells. In conclusion, findings indicate that biofabricated EMB AgNPs can combat lung cancer cells, demonstrating promising anticancer properties (114). Female breast cancer is a very prevalent kind of cancer that causes the loss of millions of lives annually. Jagtap *et al*. conducted a study showing that harnessing the phytochemical ingredient EMB from *E. ribes* fruits to produce silver NPs offers promising results as an effective treatment for cancer. The molecular docking technique reveals the potential mechanism of action. EMB has been discovered to have the potential to serve as a modulator against breast tumors that are positive for both ER and HER2 (115).

Ahmed *et al*. formulated EMB infused with silver NPs as a potential treatment against *Acanthamoeba castellanii*, the pathogen responsible for severe encephalitis and vision-threatening keratitis, due to the ongoing difficulties in existing therapies. In amoebicidal experiments, both compounds demonstrated effective reduction of *Acanthamoeba* viability. EMB exhibited an IC_50_ value of 27.16 ± 0.63 μM, while EMB-AgNPs had an IC_50 _of 13.63 ± 1.08 μM. Furthermore, both compounds exhibited minimal toxicity toward HaCaT cells in an *in vitro* study. The findings demonstrated that both samples triggered apoptosis via the mitochondria-mediated pathway. Examination of differently expressed genes revealed 652 genes with exclusive expression in treated cells compared to untreated cells. Out of them, 191 genes showed substantial regulation in the negative control group when compared to the conjugate group. The KEGG study revealed strong correlations between most genes and several biological processes, such as apoptosis, oxidative stress signaling, cytochrome P450, Rap1, and oxytocin signaling pathways. This work provides a solid basis for the creation of therapeutic drugs using EMB and EMB-AgNPs to treat microbial illnesses (116).

It takes the cooperation of governments, businesses, individuals, and international organizations to address and reduce mercury pollution in the environment. Efficient approaches encompass the implementation of regulations to restrict the release of mercury, advocating for the use of alternatives that do not contain mercury, guaranteeing the appropriate disposal of products that contain mercury, undertaking initiatives to clean up and restore affected areas, increasing public knowledge about the dangers of mercury pollution, encouraging international cooperation, and regularly monitoring the situation. However, it is essential to investigate alternate methods, such as the application of NPs. Rajalakshmi and colleagues synthesized silver NPs using the naturally produced EMB molecule. The present research showcased the efficacy of EMB NPs in efficiently eliminating mercury at a reasonable cost. EMB AgNPs successfully facilitated the solar photocatalytic degradation of the hazardous metal mercury within 60 min (117). Chemists and researchers have shown significant interest in the green production of AgNPs in recent years. Research conducted by Dhayalan *et al*. presented a technique that is environmentally friendly, cost-efficient, quick, and simple for producing silver NPs. This approach involves employing the seed extract of *E. ribes* as both a capping and reducing agent. The AgNPs exhibited a favorable anti-oxidant capability, effectively diminishing the presence of free radicals in both the anti-oxidant and antibacterial assays. AgNPs of EMB in extract form exhibited potent antibacterial properties, effectively inhibiting the development of Gram-negative (*Escherichia coli*) and Gram-positive (*S. aureus*) bacteria. Particularly, AgNPs have shown higher efficacy against Gram-negative bacteria. This green synthesis method emerged as a cost-effective, non-toxic, and environmentally friendly substitute for traditional microbiological, physical, and chemical techniques. The findings also revealed significant cytotoxicity, which refers to decreased tumor cells employing mcf-7 cell lines (A cell line derived from human breast cancer that expresses estrogen, progesterone, and glucocorticoid receptors)(118). 

For a long time, people just thought of gold as a metal. Gold has emerged as a top material for advanced applications because of its physicochemical qualities, the advent of nanotechnology, and the discovery of NPs (119). Gold nanoparticles (AuNPs) are very significant NPs that have been extensively used in medical and non-medical fields due to their exceptional characteristics, including inertness, biocompatibility, and low toxicity. Gold (Au) may be produced on a large scale by minimizing the oxidation of Au^+1^ (aureus) or Au^+3^ (auric) to Au^0^ with the addition of a reducing agent using diverse physical, chemical, and biological techniques under varying conditions. Researchers are driven by the detrimental impacts of present synthesis methods to prioritize the development of ecologically sustainable and eco-friendly synthesis techniques using non-toxic compounds derived from natural sources, such as plant extracts, phytoconstituents, bacteria, and fungi (120). Dhayalan *et al*. synthesized gold NPs of EMB alongside silver NPs. AuNPs reduced free radicals in anti-oxidant tests and inhibited the growth of Gram-negative (*E. coli*) and Gram-positive (*S. aureus*) bacteria. AuNPs were found to be very effective against Gram-negative bacteria. The MCF-7 cell lines also exhibited significant cytotoxicity, reducing tumor cells (118). The recent worldwide rise in the occurrence and spreading of antibiotic resistance (ABR) in bacterial populations poses a significant risk to human health. The emergence of ABR is causing a reduction in the availability of effective antibiotics, which in turn requires the development of new and alternative treatments and medications. The objective of the study conducted by Khare *et al*. was to evaluate the combined effectiveness of manufactured metallic NPs that were loaded with phytomolecules and stabilized with polysaccharides against *Pseudomonas aeruginosa,* and the strains of *E. coli* were obtained from river waters. The potential combined effect of EMB-loaded chitosan-gold NPs, which were produced with ciprofloxacin, was assessed utilizing a checkerboard test and time-kill curve analysis. The NPs reduced the minimum inhibitory concentration of ciprofloxacin by a factor of 16 and 4 against multi-drug-resistant *P. aeruginosa* (PA-r) and *E. coli* (EC-r) bacteria, respectively (121).

There is a pressing need for a more environmentally friendly technology due to the significant concerns surrounding the therapeutic use of plasmonic nanostructures of silver and gold produced using traditional, harmful, and inconvenient methods. The study conducted by Sasidharan *et al.* utilizes EMB, a benzoquinone derivative known for its notable medicinal qualities, as a reducing and stabilizing agent in the production of quasi-spherical gold and silver NPs, as well as gold nanostars (122). EMB-stabilized silver NPs were synthesized using sunlight. The NPs demonstrated significant antibacterial action against *S.* *aureus* and *E. coli*. Moreover, EMB was used to produce polyhedral gold NPs, which have many facets and are composed of many twinned crystals and played a crucial role in creating EMB-stabilized gold nanostars measuring 120 nm in size. These nanostars can absorb light at a near-infrared wavelength of 800 nm. The stabilized NPs of EMB demonstrated exceptional biocompatibility with both cells and human blood. In addition, the gold nanostars demonstrated exceptional computed tomographic (CT) contrast properties and significant photothermal cytotoxicity against oral epithelial cancer cells. This study presented a novel approach to creating biocompatible plasmonic nanostructures using EMB. These nanostructures have the potential to be used as antibacterial agents, for CT imaging, and as photothermal agents.

Zinc oxide (ZnO) exhibits significant optical absorption in the UVA (315-400 nm) and UVB (280-315 nm) regions, making it appropriate for a range of uses, including gene and medication delivery, cancer therapy, biosensing, antibacterial and antifungal therapies, nanomachines that may mimic biological processes and biomaterials for tissue engineering. Additionally, ZnO can function as molecular switches in shape memory polymers. Fabricating metallic NPs using environmentally friendly methods has captivated scientists for over a decade and is now widely used in engineering and biological sciences. They are very intriguing because of their significant potential. The use of plants for the manufacture of metal NPs, namely Zinc NPs, is becoming prevalent. In a study, researchers produced an extract from seeds of *E. ribes* having EMB as a significant constituent to reduce zinc acetate to create ZnO NPs (123). SLC16A1 and SLC16A3, also known as SLC16A1/3, have considerable expression levels in cervical malignancies and are closely linked to the malignant characteristics of the cancer. The SLC16A1/3 gene controls the internal and external conditions, glycolysis, and redox balance in cervical cancer cells. Blocking the activity of SLC16A1/3 offers a novel approach to eradicate cervical cancer efficiently. You *et al*. devised a drug delivery method using Gallic acid-iron NPs to enhance the anti-cancer effectiveness of EMB. The prepared EMB-loaded NPs effectively targeted the cervical cancer marker SLC16A1/3 to modulate glycolysis and redox pathways in combination with photothermal therapy. This offers a novel approach to synergistic malignant cervical cancer treatment (124).

***EMB as a synergistic agent in combination with nanoformulations***

Combinatorial therapies refer to administering two or more medications simultaneously to address different elements of the disease’s pathophysiology. This technique can potentially change the treatment of various disorders by improving medication delivery, minimizing side effects, and targeting several disease pathways simultaneously compared to single-drug therapies. Research on producing and using nanoformulations loaded with combinatorial treatments for different disorders, utilizing at least one herbal medication such as EMB (Table 2), is now a thriving study area. For example, Quan synthesized transferrin-conjugated doxorubicin and EMB encapsulated in liposomes. He then assessed their characteristics and examined their impact on breast cancer therapy. The findings revealed that the absorption of conjugated liposomes by MCF-7 cells was 3.2 times more than that of liposomes containing doxorubicin and EMB. The MTT test and *in vitro* inhibition of tumor spheroids demonstrated a potent inhibitory impact of the dual drug-loaded formulation on the breast cancer cell line (125). A study used a heat homogenization process to generate solid lipid nanoparticles (SLNs) containing both paclitaxel (PTX) and EMB. These SLNs were developed specifically for the treatment of breast cancer. The SLN formulation, which was optimized and loaded with two drugs, had a particle size of around 300 nm. This indicates that it would be appropriate for use as a parenteral formulation. The transmission electron microscopy revealed that the SLNs exhibited a uniform and spherical morphology. The entrapment efficiencies of paclitaxel and EMB were higher, and *in vitro* drug release was better. In addition, when the MCF-7 breast cancer cell line was treated with PTX-EMB loaded SLNs, it resulted in similar levels of cell toxicity as PTX solution, a combination solution of PTX-EMB and PTX loaded SLNs that were modified with PEGylation. These findings indicate that an improved SLN formulation might hold potential as an anticancer formulation (105). In another documented research, they created and analyzed SNEDD formulation that includes EMB and gliclazide in combination. The study aimed to evaluate the antidiabetic impact of this formulation in Wistar rats. The produced SNEDDs exhibited a particle size below 200 nm, a polydispersity index within an appropriate range, indicating acceptable dispersion, and an extremely negative zeta potential, suggesting excellent stability of the formulation. The percentage of cumulative release from SNEDDs was much superior and consistent compared to suspension. The combination of EMB (30 mg/kg) and gliclazide (10 mg/kg) loaded SNEDDs showed superior efficacy in the treatment of streptozotocin-induced hyperglycemia in Wistar rats, as compared to the individual administration of the pure drugs (126).

In a recently reported study, they prepared and optimized Donepezil hydrochloride (DPL) and EMB (EMB) loaded NLCs to get the highest possible drug loading, provide safer administration via the nasal route, promote efficient absorption by neurons or cells, boost accessibility to the brain, maintain controlled release, and accomplish the intended therapeutic effect in Alzheimer’s disease. NPs having a diameter of less than 200 nm were appropriate for being taken up by axons. A low Polydispersity index (PDI) value and substantially negative zeta potential value suggest that the system was uniform and monodispersed, with consistent and stable dispersion features. An analysis using the N2a cell line recommended using a 1:1 ratio of DPL and EMB to get the highest potential synergistic impact, also, the cellular absorption study revealed a predominance of the NLCs inside the cytoplasm of the cell, suggesting that N2a cells readily absorbed them. The authors concluded that intranasal treatment with DPL and EMB-loaded NLCs might be a feasible and promising approach for treating Alzheimer’s disease (127).

## Discussion

EMB, a naturally occurring benzoquinone molecule, treats many diseases through numerous mechanisms. The suppression of Plasminogen Activator Inhibiton-1 (PAI-1) by EMB may account for its impact on various metabolic processes. These include wound healing, blood coagulation, attachment and detachment of cells, cell migration, angiogenesis, and tumor-cell invasion. The quinone moiety of EMB and the presence of the phenolic OH group at C-5 are essential to its activity. The EMB quinone derivative decreases insulin levels in the plasma by inducing the movement and activation of GLUT4 in the PI3K/p-AKT signaling pathway. By preventing lipid peroxidation, EMB helps maintain the integrity of cell membranes and protects cells from oxidative damage. EMB has anti-inflammatory and anti-oxidant properties by suppressing NF-κB pathways. EMB blocks NF-κB, a transcription factor that regulates genes linked to inflammation, cell survival, and proliferation, and induces apoptosis via activating caspases and modulating Bcl-2 family proteins.

Still, the therapeutic use of EMB has been restricted due to its inadequate solubility in water. Attempts have been undertaken to enhance the solubility of EMB through synthetic techniques, such as including nitrogen-containing heterocycles in its structure or altering the polarity of its linear chain. While these methods have led to the development of water-soluble derivatives, most exhibit little activity in treating various disorders. Therefore, using structure-activity relationship techniques, EMB may serve as a pharmacophore to synthesize its more powerful and soluble derivatives in water. Another approach to enhancing the solubility and bioavailability of EMB is the novel drug delivery systems such as polymeric nanoparticles, NLCs, liposomes, nanoemulsion, and metallic nanoparticles. 

Herbal treatments are now being developed for medication delivery systems in several institutions at the primary and clinical trial stages. The only prerequisite is to devise superior ways to guarantee the efficient administration of these medications at specific locations inside the body, ensuring that the dosage does not interfere with the current treatment regimen. In the future, utilizing herbal NPs (EMB NPs) for targeted delivery through ligands and surface alteration via conjugation may captivate research groups and provide noteworthy outcomes. Further study should focus on materials that exhibit more homogeneity in composition and drug loading and enhanced capability for drug release. The necessity to address significant advancements in using theranostic-based NPs of EMB for therapeutic and diagnostic applications should also be acknowledged. There is potential for further development in creating a combination nano drug delivery system using EMB and other phytotherapeutics, drug moieties, derivatives, or nucleic acid to enhance the treatment of many illnesses via synergistic effects.

## Conclusion

EMB demonstrates remarkable versatility as a therapeutical molecule, showing substantial potential in cardiovascular health, oncology, diabetes, and neurodegenerative diseases. Its structure-activity relationship, particularly its ability to inhibit PAI-1, highlights its importance in oncology. Additionally, its anti-oxidant and anti-inflammatory properties expand its range of applications. Incorporating EMB into nanoparticulate drug delivery systems effectively tackles the issues of in vivo efficacy. This approach utilizes many advanced drug delivery mechanisms, advancing EMB into new therapeutic frontiers and serving as a convincing model for creating synergistic formulations. EMB, at the crossroads of tradition and innovation, calls out for more research into its complex interactions and the revolutionary effects it could have on therapeutic landscapes as scientific understanding expands.
